# Supplementary figures and images for: Efficient multi-task chemogenomics for drug specificity prediction
Source: PLoS One. 2018 Oct 4;13(10):e0204999. doi: 10.1371/journal.pone.0204999 (PMC6171913; doi:10.1371/journal.pone.0204999)

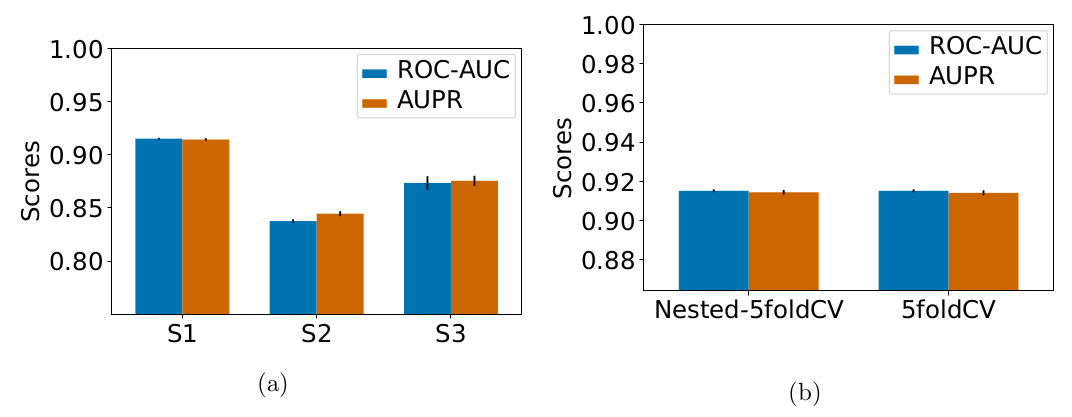

Supplement: S1 Fig — (A) Scores of MT Kernel Ridge regression on datasets S1/2/3 with a nested 5-fold-CV scheme. (B) Scores of MT Kernel Ridge regression on S1 depending on the CV scheme. (TIFF) [file pone.0204999.s001.tiff]

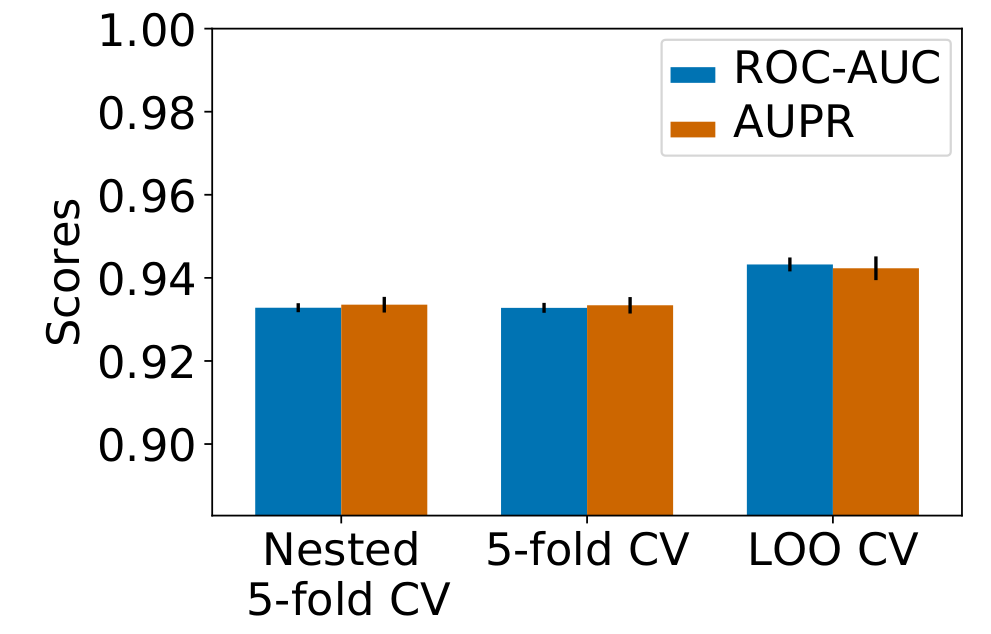

Supplement: S2 Fig — Overall, all CV schemes provide high prediction performance on this dataset, in the range of 0.93-0.94 in AUC and AUPR. The nested 5-fold-CV leads to performance very close to those of 5-fold-CV, showing that on the S1 dataset, 5-fold-CV did not suffer from overestimation of the performance due to data over-fitting. LOO-CV leads to slightly better results, although very close to those of the other CV schemes. In general, the LOO-CV scheme is expected to provide better results because the model is trained on more data points than in 5-fold-CV. Again, this problem seems to be limited here, since the performance of LOO-CV does not differ much from that of nested 5-fold-CV. (TIFF) [file pone.0204999.s002.tiff]

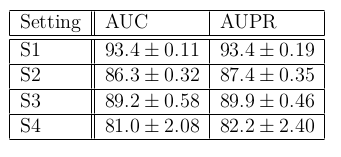

Supplement: S1 Table — (TIFF) [file pone.0204999.s003.tiff]

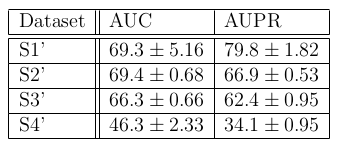

Supplement: S2 Table — (TIFF) [file pone.0204999.s004.tiff]

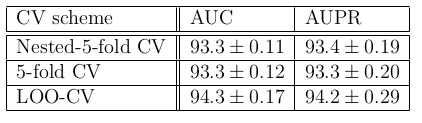

Supplement: S3 Table — (TIFF) [file pone.0204999.s005.tiff]

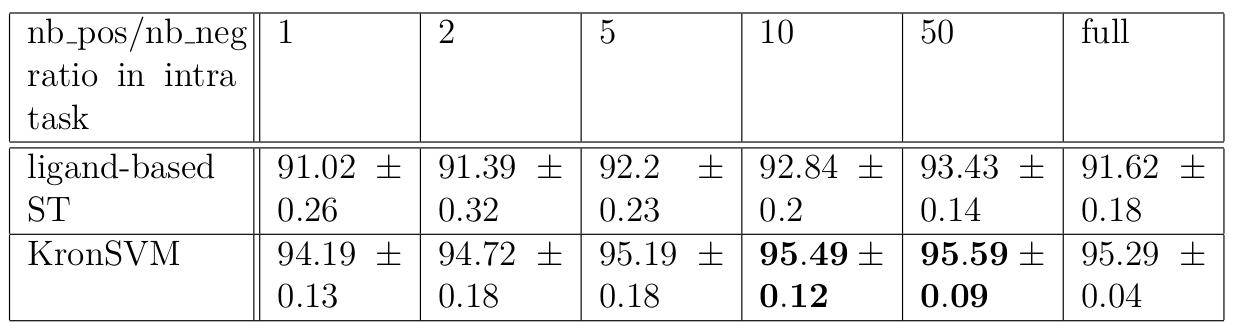

Supplement: S4 Table — (TIFF) [file pone.0204999.s006.tiff]

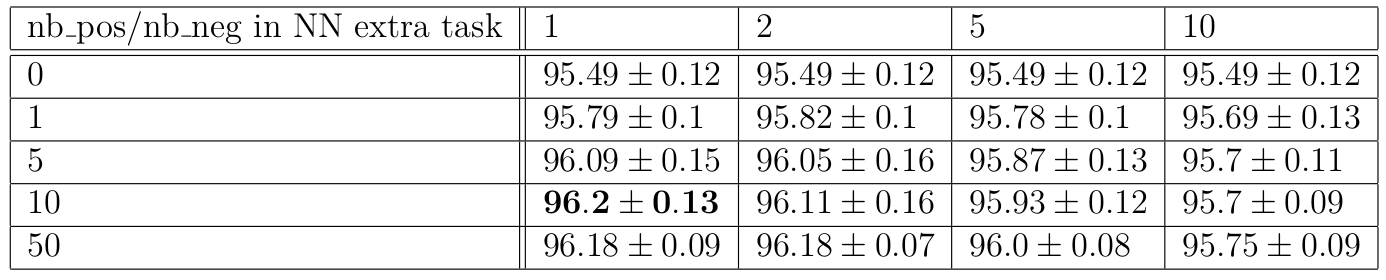

Supplement: S5 Table — (TIFF) [file pone.0204999.s007.tiff]

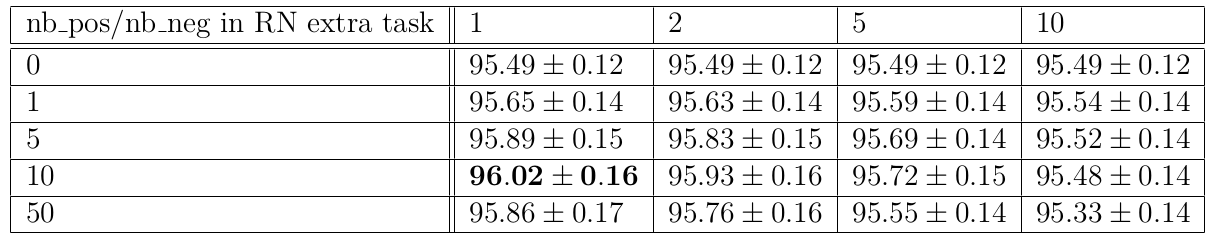

Supplement: S6 Table — (TIFF) [file pone.0204999.s008.tiff]

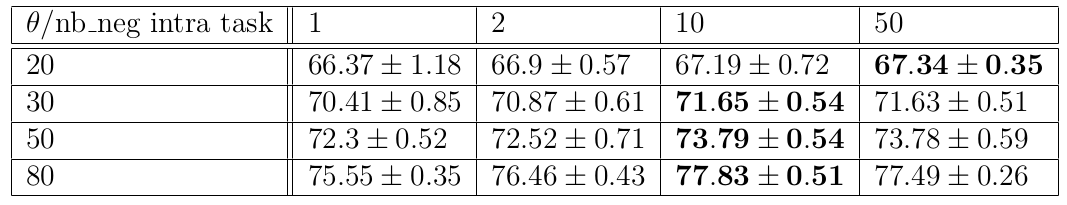

Supplement: S7 Table — (TIFF) [file pone.0204999.s009.tiff]

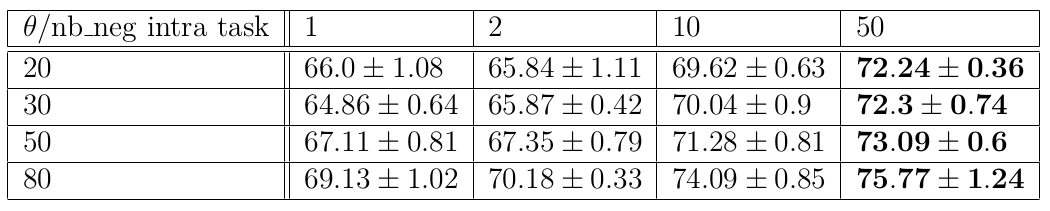

Supplement: S8 Table — (TIFF) [file pone.0204999.s010.tiff]

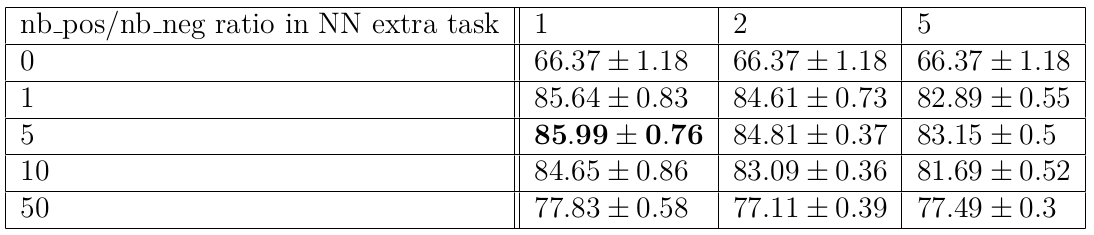

Supplement: S9 Table — (TIFF) [file pone.0204999.s011.tiff]

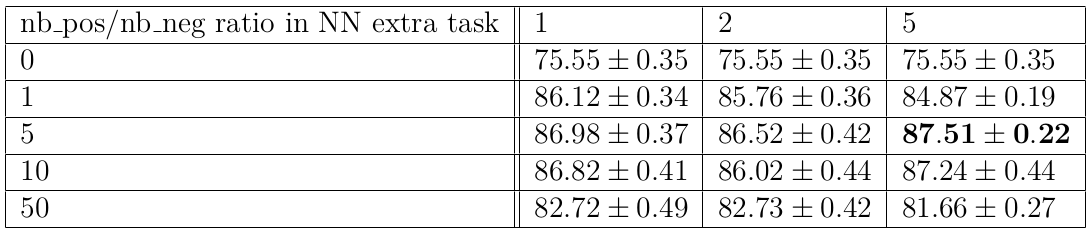

Supplement: S10 Table — (TIFF) [file pone.0204999.s012.tiff]

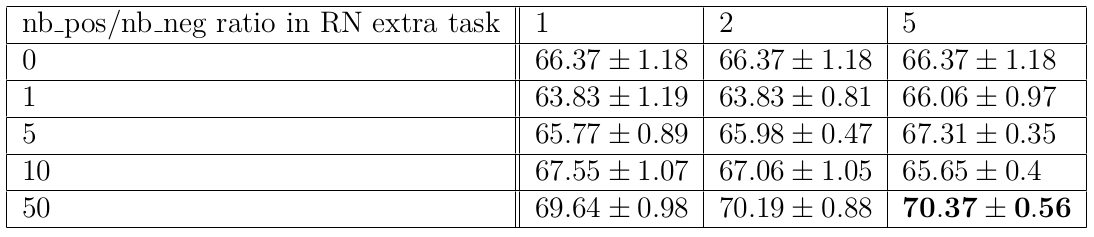

Supplement: S11 Table — (TIFF) [file pone.0204999.s013.tiff]

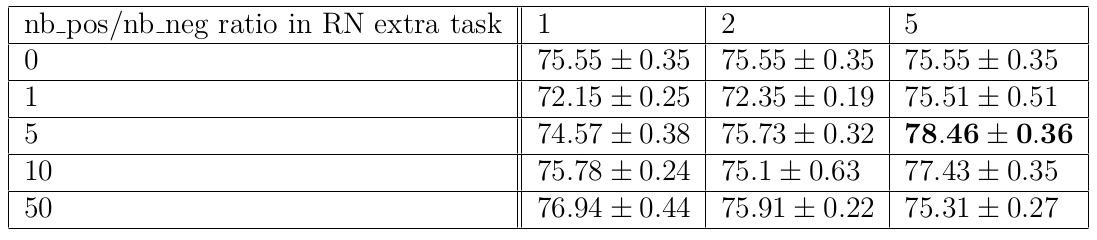

Supplement: S12 Table — (TIFF) [file pone.0204999.s014.tiff]

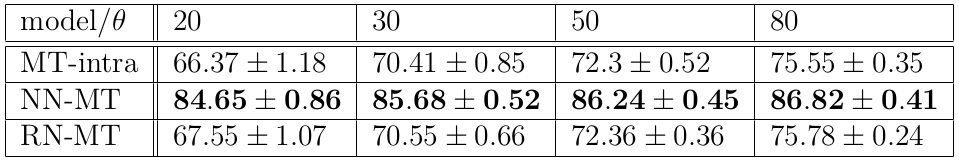

Supplement: S13 Table — (TIFF) [file pone.0204999.s015.tiff]

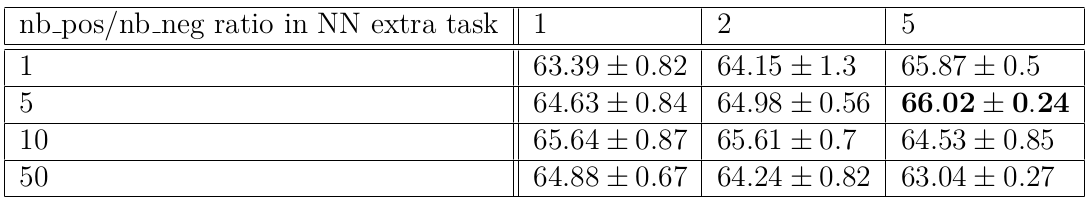

Supplement: S14 Table — (TIFF) [file pone.0204999.s016.tiff]

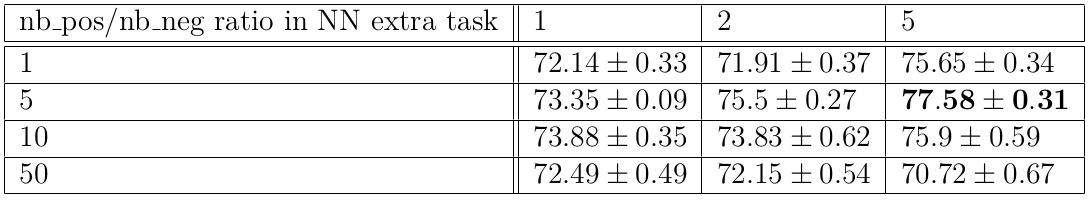

Supplement: S15 Table — (TIFF) [file pone.0204999.s017.tiff]

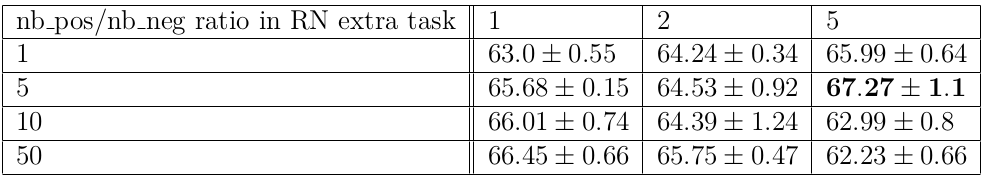

Supplement: S16 Table — (TIFF) [file pone.0204999.s018.tiff]

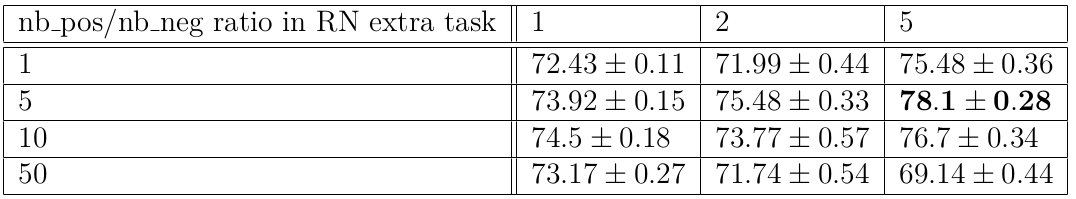

Supplement: S17 Table — (TIFF) [file pone.0204999.s019.tiff]

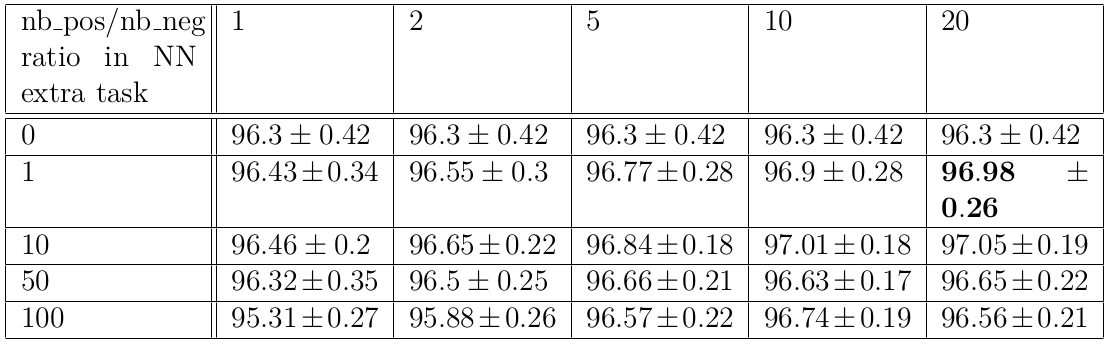

Supplement: S18 Table — (TIFF) [file pone.0204999.s020.tiff]

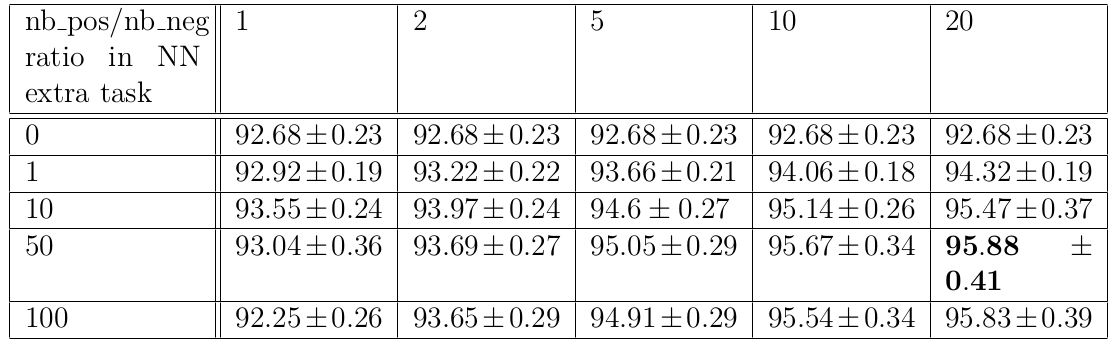

Supplement: S19 Table — (TIFF) [file pone.0204999.s021.tiff]

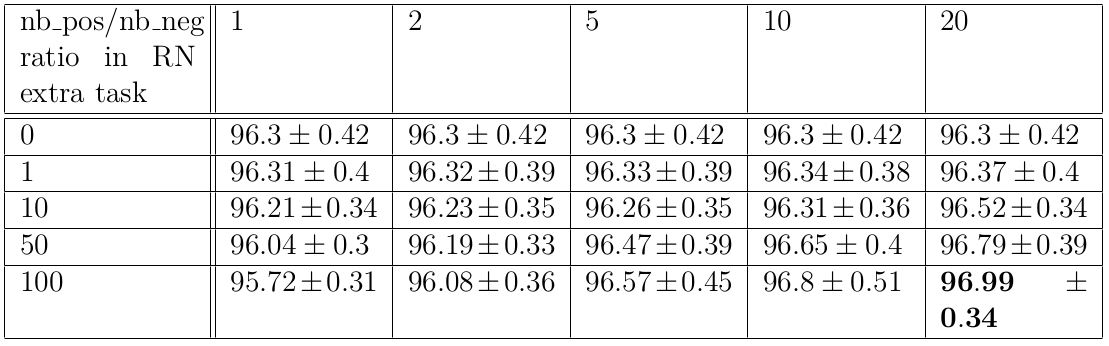

Supplement: S20 Table — (TIFF) [file pone.0204999.s022.tiff]

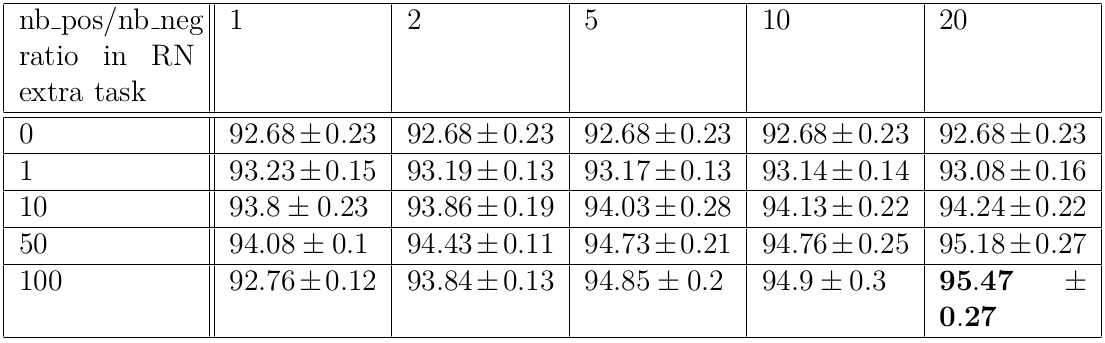

Supplement: S21 Table — (TIFF) [file pone.0204999.s023.tiff]

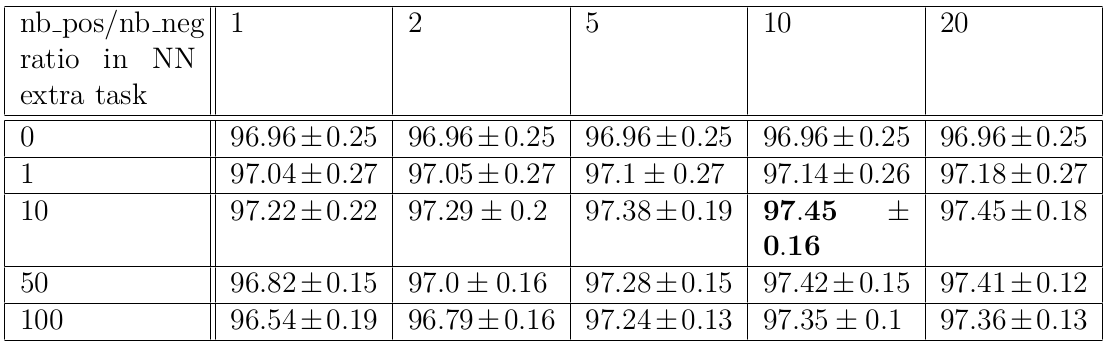

Supplement: S22 Table — (TIFF) [file pone.0204999.s024.tiff]

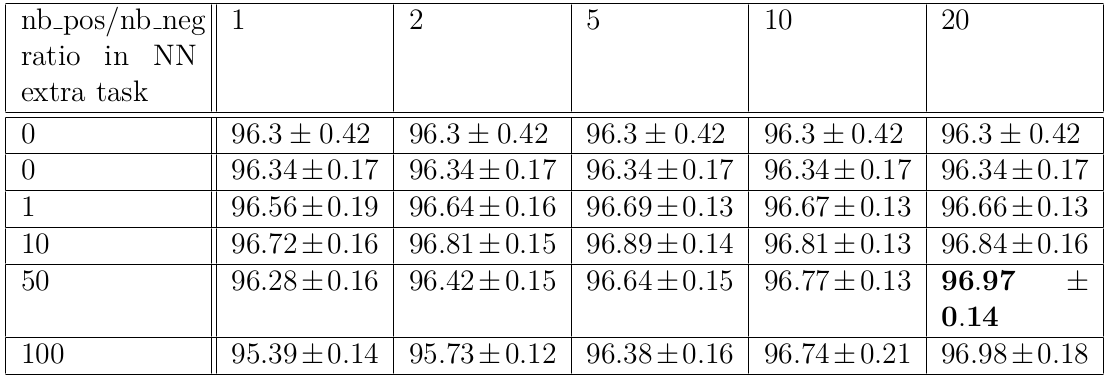

Supplement: S23 Table — (TIFF) [file pone.0204999.s025.tiff]

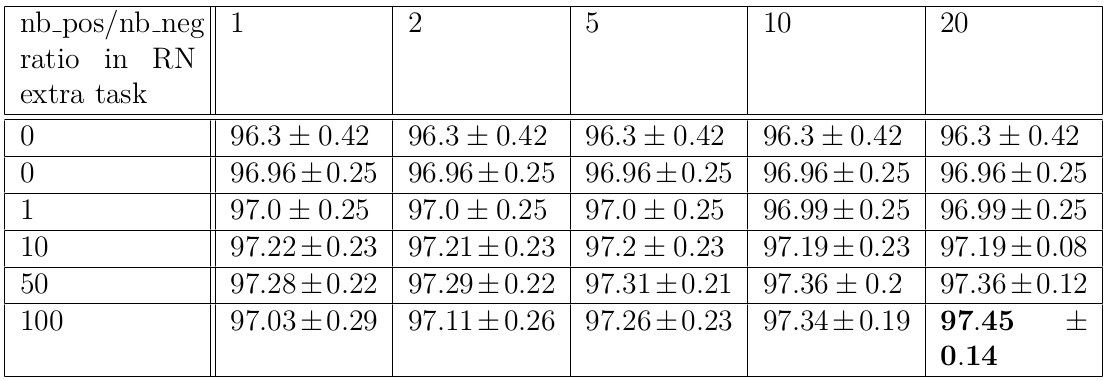

Supplement: S24 Table — (TIFF) [file pone.0204999.s026.tiff]

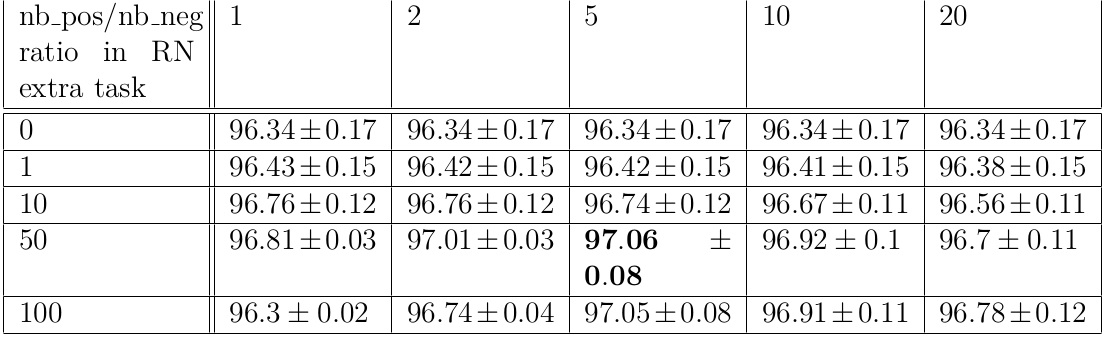

Supplement: S25 Table — (TIFF) [file pone.0204999.s027.tiff]

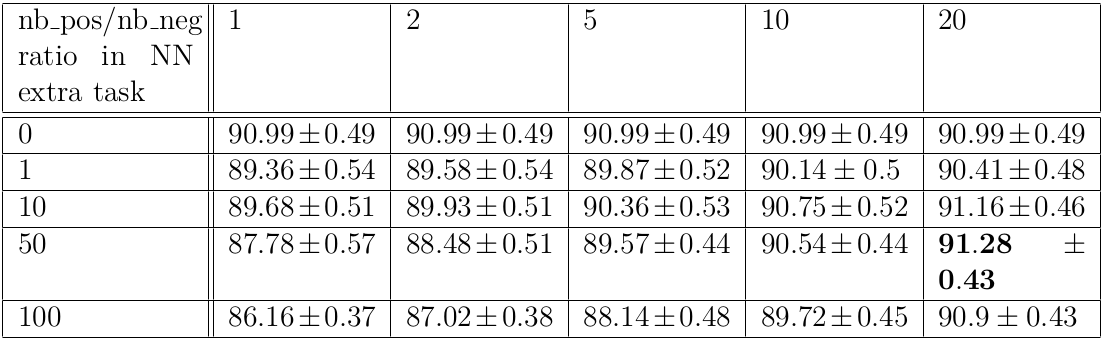

Supplement: S26 Table — (TIFF) [file pone.0204999.s028.tiff]

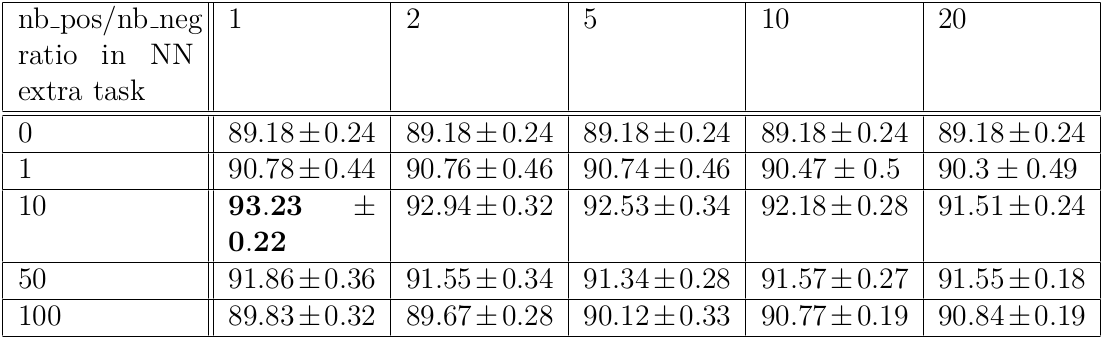

Supplement: S27 Table — (TIFF) [file pone.0204999.s029.tiff]

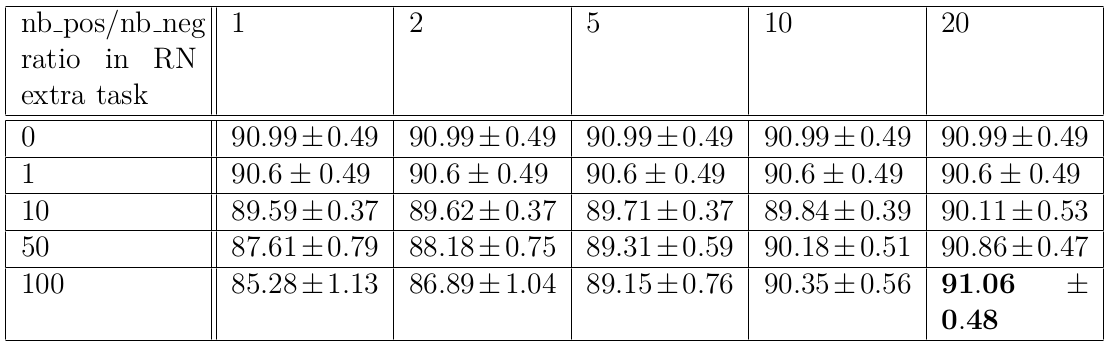

Supplement: S28 Table — (TIFF) [file pone.0204999.s030.tiff]

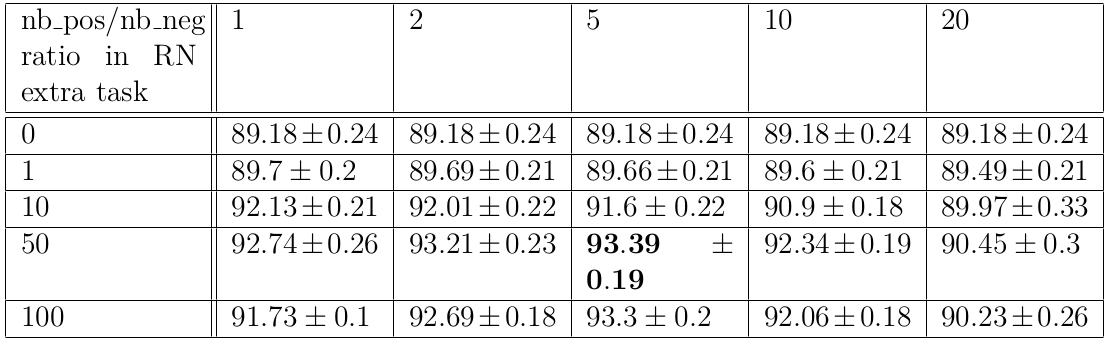

Supplement: S29 Table — (TIFF) [file pone.0204999.s031.tiff]
