# Supplementary material for: Efficient multi-task chemogenomics for drug specificity prediction
Source: PLoS One. 2018 Oct 4;13(10):e0204999. doi: 10.1371/journal.pone.0204999 (PMC6171913; doi:10.1371/journal.pone.0204999)
Supplement: S1 Appendix — (PDF) [file pone.0204999.s032.pdf]

Efficient multi-task chemogenomics for drug specificity prediction
S1 appendix: Basic principles of Support Vector Machine

Benoit Playe, Chloé-Agathe Azencott, Véronique Stoven

September 25, 2018

Let us consider a set of labeled samples S $= \{(x_1, y_1), \ldots, (x_N, y_N)\}$ where $(x_i, y_i) \in X \times \{-1, +1\}$ for $i = 1, \ldots, N$, and where the space X into which the data points live is equipped with a dot product $\langle ., . \rangle$. For example, the data points x_i represent ligands, and their labels y_i are equal to +1 for ligands that bind to a given protein and -1 for ligands that don't. In the simplest case where the two classes of data points are linearly separable, Support Vector Machines [1] (SVM) is an algorithm that learns to separate these two classes based on an hyperplane whose equation can be defined by a normal vector w and a constant b: $\langle w, x \rangle + b = 0$. Among the infinity of potential separating hyperplanes, the optimal hyperplane maximizes the margin. This margin is defined as the closest distance from the hyperplane to any of the data points. It can be shown that the search of this optimal hyperplane can be formulated by the following optimization problem:

$$\underset{w,b}{\text{argmin}} \ ||w||^2 \tag{1a}$$

$$\text{subject to } y_i \langle w, x_i \rangle + b \geq 1, \forall i = 1, \ldots, N. \tag{1b}$$

The solution hyperplane maximizes its distance to the closest data points, and this distance is equal to $2/||w||^2$.

Then, the decision function f allowing to make predictions for any new point x depends on its position with respect to the hyperplane, i.e. based on the sign of $f(x) = \langle w, x \rangle + b$.

This optimization problem is strictly convex and admits a unique solution. The Lagrangian associated to the optimization problem leads to the following

equivalent dual problem:

$$\alpha^* = \underset{\alpha}{\text{argmin}} \sum_{i=1}^{N} \alpha_i - \frac{1}{2} \sum_{i,j=1}^{N} \alpha_i \alpha_j y_i y_j \langle x_i, x_j \rangle \tag{2a}$$

$$\text{subject to } \alpha_i \geq 0, \forall i \tag{2b}$$

$$\sum_{i=1}^{N} \alpha_i y_i = 0 \tag{2c}$$

where the coefficients α_i are known as the Lagrange multipliers associated to the constraints $y_i \langle w, x_i \rangle + b \geq 1$.

In practice, this quadratic problem that can be solved efficiently using a dedicated algorithm, known as Sequential Minimal Optimization (SMO) [2]. When the optimum α^* is met, the decision function allowing to make predictions for any new point x depends on its position with respect to the hyperplane :

$$f(x) = \text{sign}\left(\sum_{i=1}^{N} \alpha_i^* y_i \langle x, x_i \rangle + b^* \right).$$

However, the two classes of data points may not be linearly separable. In these situations, kernel methods are a widely-used set of techniques that allow to adapt linear methods to non-linear models. Let us consider a semi-definite positive kernel function $K : X \times X \rightarrow R$. The Mercer theorem states that there exists a non-linear function $\phi : X \rightarrow H$ that maps data points in X into a high dimensional feature Hilbert space H where K can be expressed as a scalar product: $k(x_1, x_2) = \langle \phi(x_1), \phi(x_2) \rangle_H$.In practice, H is more often taken to be R^d. Although the two classes of data points might not be linearly separable in X, they might become linearly separable in the high dimensional space H where the SVM can be solved. The principle of kernel trick is that, since the images of the data point $\phi(x_i)$ are used only in scalar products, finding the α_i coefficients to solve the SVM can be done by replacing all occurrences of the scalar product $\langle \phi(x_i), \phi(x_j) \rangle_H$ by the kernel function $k(x_i, x_j)$:

$$\alpha^* = \underset{\alpha}{\text{argmin}} \sum_{i=1}^{N} \alpha_i - \frac{1}{2} \sum_{i,j=1}^{N} \alpha_i \alpha_j y_i y_j k(x_i, x_j) \tag{3a}$$

$$\text{subject to } \alpha_i \geq 0, \forall i \tag{3b}$$

$$\sum_{i=1}^{N} \alpha_i y_i = 0 \tag{3c}$$

In other words, finding the separating hyperplane in H does not require explicit definition of the nonlinear mapping function ϕ, or calculation of the image vectors $\phi(x_i)$.

Then, the label of a new data point x is then predicted by a function $f(x)$ defined as:

$$f(x) = \text{sign}\left(\sum_{i=1}^{N} \alpha_i^* y_i k(x, x_i) + b^*\right)$$

In the case where the two classes of points are not separable, we need to allow some of the training points to be misclassified, i.e. to be on the side of the separating hyperplane corresponding to points affected to the opposite label. To this end, we introduce a penalty terms $\epsilon_n \forall n = 1, \dots N$ (also called slacked variables) defined by: $\epsilon_n = 0$ for data points that are in the correct margin boundary and $\epsilon_n = |y_n - (\langle w, x_n \rangle + b)|$ for the misclassified points. Thus, points on the decision boundary will have $\epsilon_n = 1$, and misclassified points would be penalized by $\epsilon_n > 1$ proportionally to their distance to the separating hyperplane. Thus, the penalty terms can be written as $\epsilon_n = max(0, 1 - y_n(\langle w, x_n \rangle + b))$. Then the exact classification constraints of equation 1b are replaced by $y_i \langle w, x_i \rangle + b \geq 1 - \epsilon_i$. In addition, the penalty terms must satisfy $\epsilon_n \geq 0 \forall n = 1, \dots N$. The new objective function aims at both maximizing the margin and minimizing the penalty terms, i.e. minimizing the number of misclassified points.

$$\underset{w,b,\epsilon}{\text{argmin}} \; ||w||^2 + C \sum_{i=1}^{N} \epsilon_i \tag{4a}$$

$$\text{subject to } y_i \langle w, x_i \rangle + b \geq 1 - \epsilon_i, \forall i = 1, \dots, N, \tag{4b}$$

$$\epsilon_i \geq 0, i = 1, \dots, N. \tag{4c}$$

The parameter C in the objective function in equation 4a is meant to introduce a trade-off between the maximization of the margin, expressed by the term $\frac{1}{2}||w||^2$, and the classification error on the training set, expressed by the penalty terms. This parameter is usually determined by cross validation on the training data. In the present study, the optimal parameter C was searched between 10^{-5} and 10^5. As for the separable case, the SVM can also be solved in the non-separable case using a kernel function.

References

[1] Corinna Cortes and Vladimir Vapnik. Support-vector networks. *Machine learning*, 20(3):273–297, 1995.

[2] John Platt et al. Probabilistic outputs for support vector machines and comparisons to regularized likelihood methods. *Advances in large margin classifiers*, 10(3):61–74, 1999.
