# Supplementary material for: Efficient multi-task chemogenomics for drug specificity prediction
Source: PLoS One. 2018 Oct 4;13(10):e0204999. doi: 10.1371/journal.pone.0204999 (PMC6171913; doi:10.1371/journal.pone.0204999)
Supplement: S2 Appendix — (PDF) [file pone.0204999.s033.pdf]

Efficient multi-task chemogenomics for drug specificity prediction S2 appendix: Definition of the Kronecker product of two matrices A and B

Benoit Playe, Chloé-Agathe Azencott, Véronique Stoven

September 25, 2018

$$A \otimes B = \begin{pmatrix} a_{11}b_{11} & a_{11}b_{12} & \cdots & a_{11}b_{1q} & \cdots & \cdots & a_{1m}b_{11} & a_{1m}b_{12} & \cdots & a_{1m}b_{1q} \\ a_{11}b_{21} & a_{11}b_{22} & \cdots & a_{11}b_{2q} & \cdots & \cdots & a_{1m}b_{21} & a_{1m}b_{22} & \cdots & a_{1m}b_{2q} \\ \vdots & \vdots & \ddots & \vdots & & & \vdots & \vdots & \ddots & \vdots \\ a_{11}b_{p1} & a_{11}b_{p2} & \cdots & a_{11}b_{pq} & \cdots & \cdots & a_{1m}b_{p1} & a_{1m}b_{p2} & \cdots & a_{1m}b_{pq} \\ \vdots & \vdots & & \vdots & \ddots & & \vdots & \vdots & & \vdots \\ \vdots & \vdots & & \vdots & & \ddots & \vdots & \vdots & & \vdots \\ a_{n1}b_{11} & a_{n1}b_{12} & \cdots & a_{n1}b_{1q} & \cdots & \cdots & a_{nm}b_{11} & a_{nm}b_{12} & \cdots & a_{nm}b_{1q} \\ a_{n1}b_{21} & a_{n1}b_{22} & \cdots & a_{n1}b_{2q} & \cdots & \cdots & a_{nm}b_{21} & a_{nm}b_{22} & \cdots & a_{nm}b_{2q} \\ \vdots & \vdots & \ddots & \vdots & & & \vdots & \vdots & \ddots & \vdots \\ a_{n1}b_{p1} & a_{n1}b_{p2} & \cdots & a_{n1}b_{pq} & \cdots & \cdots & a_{nm}b_{p1} & a_{nm}b_{p2} & \cdots & a_{nm}b_{pq} \end{pmatrix}.$$

Therefore, if matrix A is of size $n.m$ and matrix B is of size $p.q$, the Kronecker product of A and B is a matrix of size $n.m.p.q$
